# EVERYDAY TECHNOLOGY: A USEFUL SERVANT BUT DANGEROUS MASTER FOR PARTICIPATION IN SOCIETY?

**DOI:** 10.1093/geroni/igz038.2849

**Published:** 2019-11-08

**Authors:** Sophie N Gaber, Sophie N Gaber, Louise Nygard, Anna Brorsson, Anders Kottorp, Sarah Wallcook, Georgina Charlesworth, Camilla Malinowsky

**Affiliations:** 1 Karolinska Institutet, Stockholm, Stockholm Lans, Sweden; 2 Division of Occupational Therapy, Department of Neurobiology, Care Sciences and Society, Karolinska Institutet, Stockholm, Stockholms Lan, Sweden; 3 Faculty of Health and Society, Malmo University.Division of Occupational Therapy, Department of Neurobiology, Care Sciences and Society, Karolinska Institutet, Malmo, Skane Lan, Sweden; 4 Research Department for Clinical, Education, and Health Psychology., London, England, United Kingdom

## Abstract

With an increasingly technological society comes an assumed ability to use Everyday Technologies (ET) in order to participate in activities and places in public space, e.g. operating ticket machines to access public transport. This study addresses a mismatch between a growing dependency on ET and evidence that people with dementia experience increased challenges using ET. The aim is to explore how ET-use and perceived risk relate to participation in public space, among people with and without dementia. People with dementia and without dementia, aged 55+, were interviewed using questionnaires including the Participation in ACTivities and Places OUTside the Home questionnaire, across Sweden (n=69) and the UK (n=128). The Swedish and UK findings show small but significant associations between total participation in places within public space, and i) ET-use, and ii) perceived risk in public space. Furthermore, people with dementia participated in fewer places within public space than those without dementia.

